# Perceptions of antenatal care services by pregnant women attending government health centres in the Buea Health District, Cameroon: a cross sectional study

**DOI:** 10.11604/pamj.2015.21.45.4858

**Published:** 2015-05-21

**Authors:** Gregory Edie Halle Ekane Edie, Thomas Egbe Obinchemti, Emmanuel Njuma Tamufor, Martin Mafany Njie, Theophile Nana Njamen, Eric Akum Achidi

**Affiliations:** 1 Department of Surgery and Obstetrics-Gynecology, University of Buea, Buea, Cameroon; 2 Department of Gynecology and Obstetrics, Regional Hospital Buea, Buea, Cameroon

**Keywords:** Antenatal care, perception, choice of site, satisfaction, Cameroon

## Abstract

**Introduction:**

User'sperception of quality of ANC services crucially impacts continuity of use of these services and hence pregnancy outcome. However in our community, ANC user's perceptions of quality are not known.

**Methods:**

An observational analytic cross-sectional study was carried out amongst pregnant women attending selected government health centres in the Buea Health District. We recruited 385 consenting pregnant women for the study. Demographic and clinical data were collected using structured questionnaires. The data was entered into Microsoft Excel and exported toEpi-Info (Version 3.5.1) for analysis.

**Results:**

Geographical accessibility and perceived quality of care were the predominant reasons for choosing or changing a site for ANC. One third of respondents (30.1%) attended a health centre out of their catchment health area with Buea Town health centre receiving the highest proportion of women out of the health area (56.8% of attendees). Knowledge about antenatal care varied and majority of respondents (96.4%) were satisfied with the antenatal services received. However, there were elements of dissatisfaction with health centre services, poor sitting facilities, amenities, few health education talks and poor nursing skills. High educational level (high school and university) (X^2^ = 8.714; p = 0.01) and first time pregnancy(X^2^= 4.217; p= 0.04) were significantly associated with poor satisfaction.

**Conclusion:**

Policy makers should implement changes in the health care delivery system taking into account the users’ preferences, more so in the light of increasing female education in Cameroon.

## Introduction

The fifth Millennium Development Goal (MDG), “Improvement of Maternal Health” [1] has Antenatal Care (ANC) coverage or attendance as a progress indicator. ANC coverage in Cameroon in 2007 stood at 82% with 60% of pregnant women having four or more visits [2]. Maternal mortality ratio (MMR) has dropped by 47% worldwide during the past 20years. However, in Cameroon, MMR has risen by 8 %from 680 to 872 deaths per 100 000 in the past 9 years after the initial decrease [3].

Studies on ANC in low income countries have mainly focused on monitoring quantifiable data such as the number of antennal visits during ANC [3, 4] and its effects on pregnancy outcome [5]. However, the perspective is changing from one of not just needing any service to one in which quality is a key component in health care provision [6, 7]. In Cameroon, there are few reported studies on the quality of ANC services rendered; meanwhile the study of consumer perceptions in relation to behavioral outcomes has been on the rise in other countries [8]. This study is therefore designed to investigate pregnant women's perception of quality of ANC services rendered in health facilities within the Buea Health District area.

## Methods

The cross sectional study was carried out in the Buea Health District, one of the 18 health districts in the South West Region of Cameroon with an estimated population of 86,272 inhabitants [9]. The health district has a total of 25 health facilities which are inequitably distributed in terms of population coverage over the 7 health areas. Antenatal care services are offered in all the government and in some of the private Health facilities. Nine integrated public health centres which accounted for about two thirds of the total study population were selected as study sites. The health centres are run by senior nurses or midwives (Head of centres) with the exception of Muea health centre which is headed by a medical doctor. The study population were pregnant women attending ANC facilities in any of the selected sites in the Buea Health District.

Ethical clearance was obtained from the Faculty of Health Sciences’ Institutional Review Board (IRB) of the University of Buea, administrative Clearance from the Regional Delegation of Public Health of the South West Region in Buea and from the heads of the different health centres involved in the study. All participants provided written or verbal informed consent. With the approval from the IRB, pregnant women aged less than the legal age of consent in Cameroon (21 years) and attending antenatal care services were considered emancipated minors and thus allowed to provide consent for themselves. Apart from the inconvenience of taking time to answer the research questionnaire, participants were not exposed to undue risk.

Confidentiality of the respondents was observed by coding questionnaires and the database was password-protected. The women benefitted from knowledge about their care and positive notions about ANC after their interview. Pregnant women who fulfilled the inclusion criteria (pregnant women presenting for the second or subsequent visit) and consented to participate in the study were enrolled into the study. Women presenting for other services than ANC visits were excluded on the grounds that they did not have any experience of a particular health centre and so could not give an opinion on the ANC services.

Between 15^th^ November 2011 and 15^th^ July 2012, a total of 385 women were recruited into the study based on the calculated sample size using the Eng's formula [10] assuming that 50% of the women were satisfied with ANC offered them. Volunteering women reporting for ANC planned visits at the study sites were sensitized on the purpose and the procedure of the study. Those who consented and signed the content form were enrolled into the study. Data was collected using a pretested interviewer-administered semi-structured questionnaire modeled according to that devised by the WHO in assessing women's perceptions about quality of care [11] and after due consultation on questionnaire design [12].

The responses in the questionnaires were transcribed into EPIInfo version 3.5.1 database (CDC/WHO, Atlanta, USA). Questionnaire data were systematically checked for errors during data entry by using legal values and specified ranges in Epi-info. In addition, 10% of the questionnaires were doubled-checked by a co-investigator, different from the original data entry person. Prior to proper analysis, the frequencies and ranges of every variable were verified for consistency with the study population. Data analysis was done using EPI info version 3.5.1. Software.Socio-demographic data was expressed using descriptive statistics and means (± Standard Deviation). Perceptions about the knowledge of ANC and the service ratings for quality were compared among these different groups using the Chi-squared test to determine associations and where it was not valid, the Fisher's exact test was used. The level of significance was designated at P

## Results

### Characteristics of respondents

**Figure 1:**
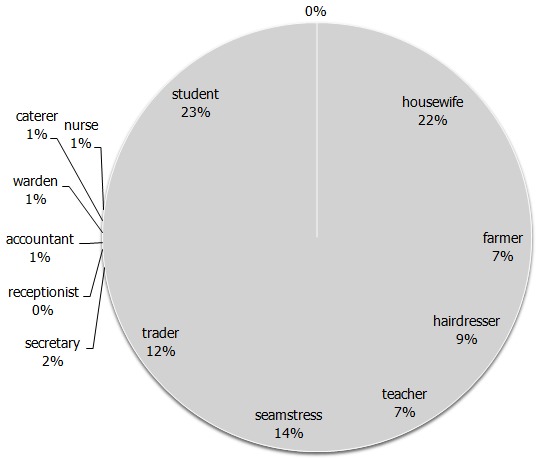
Percentage of distribution of respondents’ occupations

A total of 385 participants were recruited into the study out of 399 women who were approached for informed consent. Fourteen women withheld consent and declined to participate in the study partly because they did not have time or they did not want to answer any questions. The characteristics of the study participants are summarized in (Table 1). The mean age of the respondents was 25.2 years (SD 5.1). One third of respondents were single (n=113). The pregnancies ranged from 1 to 7, with women with the first pregnancy constituting the highest population. Most of the women were attending their second consultation and two women were at their ninth visit. Very few women (6%) enrolled for ANC during their first trimester of pregnancy, the median gestational age was 28 weeks (range 15 to 41 weeks). Most of the respondents were in secondary school (n= 141, 36.5%). Respondent's occupations varied with the highest proportion consisting of students (n = 87, 22.6%) (Figure 1).

**Table 1:** Characteristics of the study population

Characteristic		Frequency n = 385	Percentage (%)	Mean ±SD
Age group (years)	15 – 20	71	18.4	25.2 ±5.1
	21 – 25	157	40.8	
	26 – 30	98	25.5	
	31 – 35	42	10.9	
	36 – 40	17	4.4	
Marital Status	Married	272	70.6	
	Single	113	29.4	
Number of Pregnancy	1	152	40.8	
	2	102	26.5	
	3	58	15.1	
	4	42	10.9	
	≥5	16	6.8	
Number of visits	2	127	33.0	
	3	96	24.9	
	4	58	15.1	
	5	59	15.3	
	≥6	45	11.7	
Trimester at first visit	First trimester	0	0	30.3 ±5.6
	Second trimester	305	79.2	
	Third trimester	80	20.8	
Educational level	Primary	106	27.5	
	Secondary	202	52.5	
	Tertiary	77	20.0	

### Area of residence and reasons for choice of site

**Figure 2:**
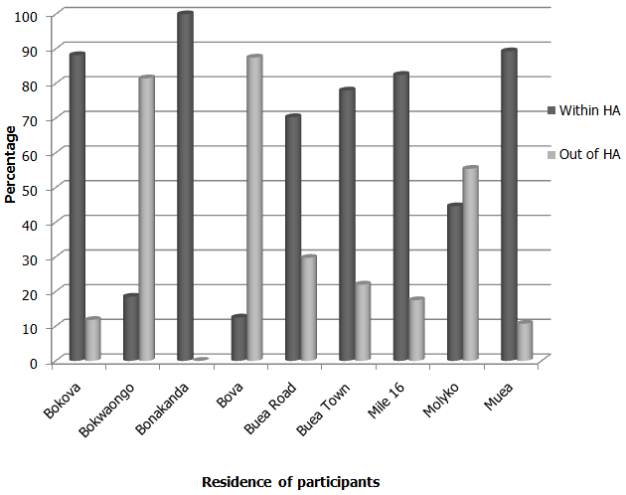
Percentage of respondents attending a particular health centre with respect to their health area of origin

The most attended health centres were those of Buea Road, PMI (“Prevention Maternelle et Infantile”), Muea and Buea Town (32.5%, 26.2% and 19.2% respectively). Meanwhile, the least attended were Bonakanda and Bova health centres (0.5% and 0.3% respectively). Two hundred and seventy (69.9%) respondents attended ANC at the health centre corresponding to their catchment health area while 30.1% attended at a different health centre out of their catchment health area (Figure 2).

The respondents’ reasons for attending ANC at their respective health centreswere accessibility, recommendation from colleague or friend, cleanliness of centre and presence of a doctor amongst others (Figure 2).Out of the 228 respondents attending subsequent ANC visits(2 or more), 127, (55.2%)of them changed their initial site of ANC visit.

### Perceptions of pregnant women

The respondent's knowledge about ANC was evaluated using three questions. Ninety-nine percent (99%) of the respondents affirmed that ANC was important not only for the mother but for the foetus as well. The other two questions concerned the timing of the first visit and the total number of ANC visits. These responses were compared to the socio-demographic characteristics; marital status, age groups, number of pregnancy (primigravidavs?2 pregnancies), number of visits and educational level (Table 2). Women who had attended antenatal visits in their previous pregnancy thought that it was beneficial to start ANC early in pregnancy unlike those who did not have this experience and who opted for third trimester enrollment.

**Table 2:** Comparison of socio-demographic characteristics with respondent's knowledge about when to start ANC visits and the number of visits

	Socio-demographic characteristic	Fisher's exact test (N = 385) = x	*p* -value
When to start ANC	Marital status	2.22	0.55
	Age group	16.56	0.09
	Pregnancy number	10.23	0.46
	Number of visit	8.13	0.039+
	Educational level	7.69	0.27
How many visits to attend	Marital status	7.8	0.049+
	Age group	36.04	0.000+
	Pregnancy number	18.97	0.000+
	Number of visit	1.075	0.81
	Educational level	11.32	0.065

+ statistically significant

Primigravida, younger and single women were less likely to know how many ANC visits they were expected to attend during their gestational period when compared to older and multiparous womenPTable 2).

### Knowledge of ANC activities done at the ANC

**Figure 3:**
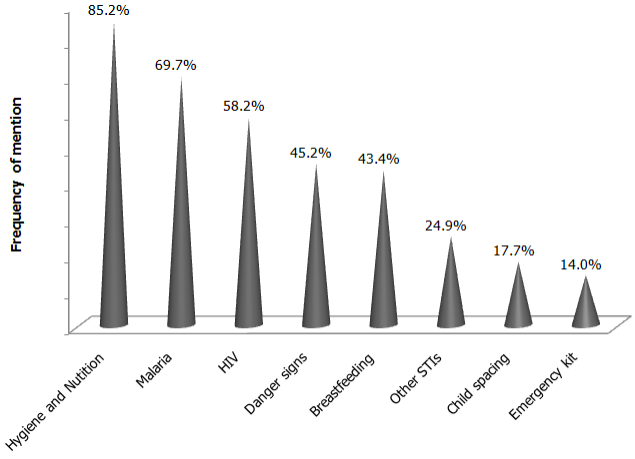
Frequency of distribution of health topics discussed during ANC

The responses by participants concerning routine activities at antenatal clinics revealed that physical examination was the most frequent activity and the least was vaccination. With respect to health education, the most mentioned topic was “hygiene and nutrition” followed by “malaria prevention” (Figure 3). The satisfaction of respondents with some of the services provided at the health centres (reception, sitting area comfort, waiting time, staff rapport, competence of personnel and comprehensiveness of health talks) was compared with the respondents’ socio-demographic characteristics to elucidate the degree to which the latter affected satisfaction. There was no statistical significance observed when respondents’ maritalstatus and age were compared with the satisfaction of the different services in the health centres using the Fisher's exact test (Table 3).

**Table 3:** P-Value of the different service satisfaction rating with respect to the educational level in the study population

Services	Fisher's exact test	p - value
Reception	0.091	P > O.O5
Sitting area comfort	0.842	
Waiting time	4.357	
Staff rapport	1.838	
Competence of personnel	0.672	
Health talks	5.43	

### Pregnancy groups

There was a statistically significant association when comparing respondents at their first pregnancy with those at their second or more pregnancies in terms of their satisfaction ratings; the sitting area comfort (F= 7.052; P = 0.04), competence of staff (F = 8.92, P = 0.02) and health talk comprehensiveness (F = 18.499, P

### Number of Visits at the time of study

There was no statistically significant difference when the service rating (reception,sitting area comfort, waiting time, staff rapport, competence of personnel and health talk) of women attending their second visit and those attending subsequent visits was compared.

### Educational level

There was insignificant difference using the Fisher's exact test, with regards to all the service ratings when different educational levels were compared (Table 3). However, women with a high level of education were less satisfied with the reception as compared to those with a low educational level. Furthermore, they were less satisfied with the comfort in the sitting area and the way the staff attended to them. Respondents of low educational level tend to be very satisfied with the personnel's competence and they also rated the health talks as very satisfactory, in marked contrast to those of a higher educational level who were much more likely not to be satisfied with the health talks.

### Overall satisfaction

Most of the women were satisfied with the care rendered them at the different health centres with 96.9% being “very satisfied”. The overall client satisfaction was compared with the different socio-demographic characteristics of the respondents (Table 4). Only the pregnancy grouping and the educational level of respondents was associated with a statistical significant difference in terms of their overall satisfaction. Women at their first pregnancy and those with a high level of education were more likely to be unsatisfied with the ANC services they received at their attending health centres.

**Table 4:** Comparison between overall satisfaction and socio-demographic characteristics of the study population

Socio – demographic characteristics		Level of satisfaction		X^2^; P-value
		Satisfied (%)	Not satisfied (%)	
marital status	single	99 (87.6)	14 (12.4)	1.99; p = 0.159
	married	218 (80.1)	54 (19.9)	
Age group	15 – 20	61 (85.9)	10 (14.1)	5.66; p = 0.226
	21 – 25	125 (79.6)	32 (20.4)	
	26 – 30	87 (88.8)	11 (11.2)	
	31 – 35	36 (85.7)	6 (14.3)	
	36 – 40	14 (82.4)	3 (17.6)	
Pregnancy group	primigravida	138 (87.9)	19 (12.1)	4.217;p = 0.04
	2 –7 pregnancies	214 (93.6)	14 (6.4)	
Number of visit	2	105 (82.7)	22 (17.3)	1.249; p= 0.33
	3 – 9	222 (86)	36 (14)	
Educational level	Primary	98 (92.5)	8 (7.5)	8.714;p = 0.01
	Secondary	190 (94.1)	12 (5.9)	
	Tertiary	64 (83.1)	13 (16.9)	

### Recommendations from the study participants

Of the 385 respondents, less than half, 42.3% (163) proposed a recommendation to ameliorate or improve service delivery by the health centres. These recommendations were pooled into themes of which the most prevalent theme concerned health centre amenities (20.2%) such as good toilet facilities, cleanliness, reduced congestion and preferential care towards the women in aspects such as rapid delivery of laboratory results. The respondents also wished that the health talks (16.3%) should be more regular and that primigravida should be encouraged to come regularly. Women of high educational level were the most interested in the health talk recommendation (76% of all recommendations were made by this class of respondents), arguing that the “lectures” should be more detailed than what they were at the moment.

## Discussion

The study aimed at exploring the perceptions of pregnant women attending antenatal clinic in the public health centres in the Buea Health District regarding antenatal care in terms of their knowledge and satisfaction with service delivery. It also sought to establish reasons that determined these women's choice of ANC site as well as explore the geographic distribution of the women with respect to their residence and their attending health centre.

Only 6% of women started visits in the first trimester while 14.5% began in the third trimester. This observation is similar to studies carried out in Kenya [13] and South Africa [14] where women started clinic late because of perceived lack of benefit, limited facility access, uncertainty about pregnancy and “laziness” to go for many visits. Late antenatal visits attendance is detrimental because it prevents the clients from obtaining the maximum benefits from the antenatal service [14–16].

With respect to the reasons that influenced the respondents’ choice of health centre, the main factor affecting the choice of ANC site was geographical accessibility. This observation is similar to that seen in Uganda [17] and Zimbabwe [18] where it was also shown that the quality of care provided was contributory. Some of the respondents (25.6%) were recommended an ANC site. Of these, 49.6% recommendations were made by friends and neighbors (12.4%) mother or sister (12.4%) and husband (10%). This illustrates the importance of previous contact with a facility to determine further use. Here, the perception of the past users is the only driving tool to refer. The husband has been shown to play a significant role in determining the choice of ANC site by the woman in a Nigerian [19] and Ugandan study [20] where they accounted for 43% and 32% of all recommendations respectively. Evaluation of the reasons why 55.2% of respondents changed their ANC site, revealed that the change of residence, closeness to the health facility and the quality of care offered at the previous site were the predominant reasons. This further emphasizes the importance of distance as a dominant factor in determining women's choice of site [19].

One fifth of the participants were dissatisfied with the amenities that were provided at some of the health institutions. Hence efforts should be geared towards improving on health centre variables (cleanliness, comfort in sitting area and quality of care amongst others) to improve on their utilization by the women who live nearby especially for those health centres which are relatively new and by so doing increasing their attendance rate.

Pregnant women's knowledge about the timing, goals of ANC visits, and the number of antenatal visits was very high among the study participants contrary to what was shown in studies carried out in Tanzania [14] and South Africa [15]. Concerning the number of ANC visits, the health centres follow the Western model of clinic visits with an average of 10 to 12 visits. However, attitudes are changing towards the “minimum of 4 visits” recommended by the WHO after the work of Nigenda et al [21] and Langer et al. [6] in resource limited settings like the study area. If this approach is vulgarized, it might increase the uptake of ANC services and improve on the quality of health care.

The health care education topics were similar among the studies reviewed though there were some disparities in the frequency. The topics focused mainly on care in pregnancy (diet, exercise and importance of ANC) and for the newborn (diet and breastfeeding). The most discussed health topic was hygiene and nutrition in pregnancy like in other studies [4, 22]. 45.2% of respondents received information on the detection or management of pregnancy danger signs, an observation similar to the 42% in Pembe's study in Tanzania [23]. In marked contrast, the Kenyan study by Ouma and collaborators [24] and that in Nigeria by Dairo and Owoyokun [25] found that only 10.4% and up to 89.6% of pregnant women were informed of pregnancy danger signs respectively. In our setting, family planning and care of the newborn are usually discussed in Infant Welfare Clinic. This accounts for the low rate of these health talks in this study as opposed to other studies [26].

Respondents rated the services provided at the health centres generally as satisfactory. The activities carried out in the health centres were similar in the institutions involved in the study. The services offered were acceptable in contrast with observations from other studies in Sub-Saharan countries such as in Uganda by Cham et al [27] and that by van Eijket al [4] in Kenya where for example, urine and serological tests are not routinely done. In a Nigerian study by Fawoleet al [7] and in Entebe, a semi-urban district in Uganda [28] a proportionately similar profile of ANC services were offered as in our area of study.

With regards to waiting time at the clinic, the women recognized that most of the time wasting was due to late coming of their fellow pregnant women. However, dissatisfaction abounded with respect to this aspect, especially for women of higher educational levels. They were impatient because they claimed they had other preoccupations. Furthermore, respondents had some reservations with respect to the sitting comfort in some health centres, complaining that the benches were too hard and lacked back-rests. The women with high level of education and women attending for their first pregnancies were not satisfied with the competence of the staff. Most of them recommended an improvement in the nurses’ skills. However, women attending for their subsequent pregnancies and those at their third or more visits were more tolerant of the services provided at the health centres. This is probably because their contact with the health system had tailored their expectations and they expressed satisfaction with the existing services.

There were significant differences between primigravida or multigravida and the educational levels on the aspect of the comprehensiveness of health talks. Women attending ANC for their subsequent pregnancies probably had a notion of what health topics were discussed during clinic sessions and so their objectives at ANC were not only aimed at acquiring knowledge about diet, danger signs and other topics but also in the state of their babies. Primigravida on the contrary expected vital information from the health talks to help them cope well with their pregnancy and labor [29]. Clients with a high educational level, were less satisfied with the health talks. They were more interested in having an indebt knowledge on how complications arising during pregnancy could be managed. The predominant topics discussed during health talks were hygiene and nutrition. There were post-natal lectures on the immediate care of the newborn and breastfeeding. Focusing on post-natal issues was not usually appealing to pregnant women who were more eager to learn about labor. However, an Australian study showed that both women and providers thought the time for antenatal classes was too small to incorporate all they had to learn with some women complaining of having received too much information [29].

The overall satisfaction of the women attending ANC in the study health district was high(91.4%). These results are similar to other studies of patient satisfaction; (96.5%) in Ibadan, Nigeria [7] and 86.4% in Ogun State, Nigeria [25]. However, it has been said that the views of pregnant women concerning their care is generally positive as they tend to be uncritical of healthcare, accepting whatever care they receive as appropriate [29]. Thus using patient views to evaluate health care has been criticized [30], regarding their responses as a mixture of the healthcare performance measurement and the respondent's reflection. Nevertheless, patient views provide an insight to the care provided and the system as a whole. The influence of the educational level of respondents is predominant here as it is noticed that those at a high educational level are more likely to be critical about care received and defer a positive satisfaction. This issue was also revealed in Fawole and colleagues’ study [7] where they hypothesized that as the level of education in the community steadily increases, pregnant women may become more and more critical of health care. Hence there is a need to mobilize efforts for a better quality assessment in our health care provision with the aim of improving quality in terms of provision of health care services. Improvement must be made to attain a desired change and amelioration in our health care delivery package. The users’ opinion has been sought here and it is noticed that they recommend ameliorations in the interpersonal interaction with staff in terms of more comprehensive health talks and improvement in nursing skills, better infrastructures(like good toilet facilities and comfortable sitting areas) and tolerable waiting times. A step in this direction will go a long way to consolidate the improvement in health care delivery sought by Cameroon in the framework of the MDGs.

## Conclusion

About one third of pregnant women in our study population attended an ANC site different from their local catchment area despite the availability of ANC in their residential area. Geographical accessibility was the main reason for choosing an ANC site, followed by perceived quality of care provided. Pregnant women in the Buea Health District have different knowledge about ANC which varies significantly among the pregnancy groups, number of visits and age. Most pregnant women were satisfied with the level of care received during ANC in public health centres in the Buea Health District. High educational level and primiparity are negatively associated with pregnant women's satisfaction of care received at ANC. Taken together, policy makers should implement changes in the health care delivery system taking into account the users’ opinion, more so in the light of increasing female education in Cameroon.

## References

[1] United Nations (2010). The Millennium Development Goals Report. http://www.who.int/topics/millenniumdevelopmentgoals/maternalhealth/en/.

[2] World Health Organization (2010). Trends in maternal mortality 1990 – 2010.

[3] (2012). Demographic Health Survey.

[4] VanEijk AM, Bles HM, Odhiambo F, Ayisi JG, Blockland IE, Rosen DH, Adazu K, Slutsker L, Lindblade KA (2006). Use of antenatal services and delivery care among women in rural western Kenya: a community based survey. Reproductive Health..

[5] Mafany NM, Mati JKG, Nasah BT (1990). Maternal Mortality in the South West Province of Cameroon 1982-1987, Annuals. Universitaires Sciences Santé..

[6] Langer A, Villar J, Romero M, Nigenda G, Piaggio G, Kuchaisit C, Rojas G, Al-Osimy M, Belizán JM, Farnot U, Al-Mazrou Y, Carroli G, Ba'aqeel H, Lumbiganon P, Pinol A, Bergsjo P, Bakketeig L, Garcia J, Berendes H (2002). Are women and providers satisfied with antenatal care? Views on a standard and a simplified, evidence-based model of care in four developing countries. BMC Women's Health..

[7] Fawole AO, Okunlola MA, Adekunle AO (2008). Clients’ perceptions of the quality of antenatal care. J Natl Med Assoc..

[8] Nisar N, White E (2003). Factors affecting utilization of antenatal care among reproductive age group women (15-49 years) in urban squatter settlement of Karachi. Journal of Pakistan Medical Association..

[9] Regional Delegation of Public Health, South West Region: Ministry of Public Health, Cameroon (2010).

[10] Eng J (2003). Sample size estimation. How many individuals should be studied?. Radiology..

[11] Langer A, Nigenda G, Romero M, Rojas G, Kuchaisit C, Al-Osumi M, Orozco E (1998). Conceptual bases and methodology for the evaluation of women's and providers’ perception of the quality of antenatal care in the WHO antenatal care randomized controlled trial. Paediatr Perinat Epidemiol..

[12] Siniscalco MT, Auriat N, Ross K (2005). Questionnaire design. Quantitative research methods in educational planning.

[13] Mwaniki PK, Kabiru EW, Mbugua GG (2002). Utilization of antenatal and maternity services by mothers seeking child welfare services in Mbeere District, Eastern Province, Kenya. East Afr Med J..

[14] Myer L, Harrison A (2003). Why do women seek antenatal care late? Perspectives from rural South Africa. J Midwifery Women's Health..

[15] Fomulu JN, Tiyou KC, Mbu RE, Nana NP, Leke RJI (2008). Mortalité Maternelle à la Maternité Principale de Yaoundé: Etude Rétrospective de 2001 à 2006. Health Sciences and Disease..

[16] Hogan MC, Foreman KJ, Naghavi M, Ahn SY, Wang M, Makela SM (2010). Maternal mortality for 181 countries 1980-2008: A systematic analysis of progress towards Millennium development goal 5. Lancet..

[17] Birungi S, Odaga J, Lonchoro JP, Santini S, Owiny V, De Vivo E (2009). The Quality and Use of Maternal Health Care in Oyam District, Uganda: A baseline survey for an intervention. Health Policy and Development Journal..

[18] Kambarami RA, Chirenje MZ, Rusakaniko S (1999). Antenatal care patterns and factors associated with perinatal outcome in two rural districts in Zimbabwe. Cent Afr J Med..

[19] Adamu Y, Salihu H (2002). Barriers to the use of antenatal and obstetric care services in rural Kano, Nigeria. J Obstet Gynecol..

[20] Blackwell DA (2002). Prenatal care services in the public and private arena. J Am Acad Nurse Pract..

[21] Nigenda G, Langer A, Kuchaisit C, Romero M, Rojas G, Al-Osimy M, Villar Jose, Garcia J, Al-Mazrou Y, Ba'aqeel H, Carroli G, Farnot U, Lumbiganon P, Belizán J, Bergsjo P, Bakketeig L, Lindmark G (2003). Womens’ opinions on antenatal care in developing countries: Results of a study in Cuba, Thailand, Saudi Arabia and Argentina. BMC Public Health..

[22] Mlay R, Massawe S, Lindmark G, Nystrom L (1994). Recognition of risk factors in pregnancy among women attending antenatal clinic at Mbagala, Dar es Salaam. East Afr Med J..

[23] Pembe AB, Carlstedt A, Urassa DP, Lindmark G, Nystr'm L, Darj E (2010). Quality of antenatal care in rural Tanzania: counselling on pregnancy danger signs. BMC Pregnancy and Childbirth..

[24] Ouma PO, Van Eijk AM, Sikuku ES, Odhiambo FO, Crawdord SB, Ayisi JG, Kager PA, Slutsker L (2010). Antenatal and delivery care in Rural Western Kenya: the effect of training health workers to provide “Focused Antenatal Care”. Reproductive Health..

[25] Dairo MD, Owoyokun KE (2010). Factors Affecting the Utilization of Antenatal Care Services in Ibadan, Nigeria. Benin Journal of Post GraduateMedicine..

[26] Porter M, Macintyre S (1984). What is, must be best: a research note on conservative or deferential response to antenatal care provision. Soc Sci Med..

[27] Cham M, Sundby J, Vangen S (2005). Maternal mortality in the rural Gambia, a qualitative study on access to emergency obstetric care. Reproductive Health..

[28] Chann CJ, Kizza M, Morison L, Mabey D, Muwanga M, Grosskurth H, Elliott AM (2007). Use of antenatal services and delivery care in Entebbe, Uganda: a community survey. BMC Pregnancy and Child Birth..

[29] Renkert S, Nutbeam D (2001). Opportunities to improve maternal health literacy through antenatal education: an exploratory study. Health Promotion International..

[30] Sitzia J, Wood N (1997). Patient satisfaction: a review of issues and concepts. Soc Sci Med..

